# Evaluation of Polycyclic Aromatic Hydrocarbons Using Analytical Methods, Toxicology, and Risk Assessment Research: Seafood Safety after a Petroleum Spill as an Example

**DOI:** 10.1289/ehp.1306724

**Published:** 2013-11-08

**Authors:** Jeffrey Wickliffe, Edward Overton, Scott Frickel, Jessi Howard, Mark Wilson, Bridget Simon, Stephen Echsner, Daniel Nguyen, David Gauthe, Diane Blake, Charles Miller, Cornelis Elferink, Shakeel Ansari, Harshica Fernando, Edward Trapido, Andrew Kane

**Affiliations:** 1 Department of Global Environmental Health Sciences, Tulane University, New Orleans, Louisiana, USA; 2 Department of Environmental Sciences, Louisiana State University, Baton Rouge, Louisiana, USA; 3 Department of Sociology, Washington State University, Pullman, Washington, USA; 4 Mary Queen of Vietnam Community Development Corporation Inc., New Orleans, Louisiana, USA; 5 Bayou Interfaith Shared Community Organizing, Thibodaux, Louisiana, USA; 6 Department of Pharmacology and Toxicology, and; 7 Department of Biochemistry and Molecular Biology, University of Texas Medical Branch, Galveston, Texas, USA; 8 Department of Epidemiology, Louisiana State University Health Sciences Center, New Orleans, Louisiana, USA; 9 Department of Environmental and Global Health, University of Florida, Gainesville, Florida, USA

## Abstract

Background: Polycyclic aromatic hydrocarbons (PAHs) are abundant and widespread environmental chemicals. They are produced naturally and through man-made processes, and they are common in organic media, including petroleum. Several PAHs are toxic, and a subset exhibit carcinogenic activity. PAHs represent a range of chemical structures based on two or more benzene rings and, depending on their source, can exhibit a variety of side modifications resulting from oxygenation, nitrogenation, and alkylation.

Objectives: Here we discuss the increasing ability of contemporary analytical methods to distinguish not only different chemical structures among PAHs but also their concentrations in environmental media. Using seafood contamination following the *Deepwater Horizon* accident as an example, we identify issues that are emerging in the PAH risk assessment process because of increasing analytical sensitivity for individual PAHs, and we describe the paucity of toxicological literature for many of these compounds.

Discussion: PAHs, including the large variety of chemically modified or substituted PAHs, are naturally occurring and may constitute health risks if human populations are exposed to hazardous levels. However, toxicity evaluations have not kept pace with modern analytic methods and their increased ability to detect substituted PAHs. Therefore, although it is possible to measure these compounds in seafood and other media, we do not have sufficient information on the potential toxicity of these compounds to incorporate them into human health risk assessments and characterizations.

Conclusions: Future research efforts should strategically attempt to fill this toxicological knowledge gap so human health risk assessments of PAHs in environmental media or food can be better determined. This is especially important in the aftermath of petroleum spills.

Citation: Wickliffe J, Overton E, Frickel S, Howard J, Wilson M, Simon B, Echsner S, Nguyen D, Gauthe D, Blake D, Miller C, Elferink C, Ansari S, Fernando H, Trapido E, Kane A. 2014. Evaluation of polycyclic aromatic hydrocarbons using analytical methods, toxicology, and risk assessment research: seafood safety after a petroleum spill as an example. Environ Health Perspect 122:6–9; http://dx.doi.org/10.1289/ehp.1306724

## Introduction

In the past several decades, there have been numerous petroleum leaks from transport vessels, pipelines, and exploration wells. Production-well accidents have also resulted in several large spills. Natural seepage of crude oil also contributes to the petroleum load in the environment (Farwell et al. 2009). Spills and leaks in coastal areas and adjacent marine environments can negatively impact marine and coastal biota and increase concern regarding potential health effects in cleanup workers and residents. Contamination of locally harvested seafood species with toxic polycyclic aromatic hydrocarbons (PAHs) represents a major concern because consumption is a major route for human exposure (Dickey 2012; Gohlke et al. 2011; Goldstein et al. 2011; Peacock and Field 1999; Rotkin-Ellman and Solomon 2012; Rotkin-Ellman et al. 2011; Xia et al. 2012).

With respect to seafood caught or harvested in spill or contaminated areas, subsistence, recreational, and commercial fisheries are invariably impacted. Local, state, and federal public health officials are charged with monitoring seafood safety during and following petroleum accidents to minimize possible health effects that may result from consumption of contaminated seafood. Researchers typically measure petroleum contaminants in potentially impacted coastal and marine species, including seafood, as a component of their research to estimate possible ecological or human health consequences. Debate regarding seafood safety and estimates of possible increased health risks during and following such events has often centered on key aspects of health risk models and the health risk assessment process (Dickey 2012; Gohlke et al. 2011; Rotkin-Ellman and Solomon 2012; Rotkin-Ellman et al. 2011). For example, health risk–based levels of concern for PAHs in seafood calculated by the U.S. Food and Drug Administration (FDA) after the *Deepwater Horizon* accident have been questioned as not protecting sensitive subgroups such as the developing fetus, children, and women primarily of childbearing age (Dickey 2012; Gohlke et al. 2011; Rotkin-Ellman and Solomon 2012; Rotkin-Ellman et al. 2011). In fact, little is known in the field of toxicology about the negative health effects that consumption of PAHs—especially from consumption of contaminated seafood—may have on the developing fetus, children, or adolescents. Furthermore, application of various acceptable risk levels, consumption rates, exposure duration, and estimates of body weights have been hotly debated because these metrics, in addition to measured PAH levels, drive the estimates of both cancer and noncancer disease risks (Dickey 2012; Gohlke et al. 2011; Rotkin-Ellman and Solomon 2012; Rotkin-Ellman et al. 2011).

PAHs primarily consist of carbon and hydrogen. PAHs have two primary sources: They are formed either by combustion of organic matter (e.g., forest fires, fossil fuel combustion) or by the diagenetic transformation of mostly plant material deposited deep within the earth’s crust. These sources of PAHs are known as pyrogenic and petrogenic, respectively. These formative processes do not produce a single molecular structure, but instead generate cyclic compounds with 2–7 fused benzene rings in different configurations. There are hundreds of different PAHs; however, the composition of PAHs from combustion is noticeably different from that of PAHs produced by diagenetic processes (PAHs found in crude oil, coal, or shale, for example). Pyrogenically produced PAHs are primarily compounds with unsubstituted aromatic rings; these PAHs are often called parent PAH structures. Petrogenically produced PAH compounds have alkyl group substitutions on the various parent ring structures. A small fraction of the PAHs found in petrogenic sources include unsubstituted or parent compounds. Therefore, analytical data that include information on alkyl substitution makes it relatively easy to determine whether the PAHs in an environmental sample are from pyrogenic or petrogenic sources, or are a mixture of both.

Contemporary analytical methods are increasingly able to distinguish not only different chemical structures among PAHs but also their concentrations in environmental media. Using seafood contamination after the *Deepwater Horizon* incident as an example, we describe the emerging issues in the PAH risk assessment process due to increasing analytical sensitivity for individual PAHs and the paucity of toxicological literature for many of these compounds.

## Discussion

Analytical chemical methods designed to determine the compositional nature and quantity of specific PAHs in environmental and biological media have progressed substantially in the last 10 years. Contemporary methods using gas chromatography (GC) followed by mass spectrometry (MS) under selective ion monitoring (SIM) modes can now discriminate hundreds of specific PAH compounds, as well as their individual quantities in complex biological samples. For example, advanced methods using highly automated and efficient extraction protocols coupled with GC and quadrupole MS rapidly identify and quantify classic unsubstituted PAH analytes (UPAHs; e.g., benzo[*a*]pyrene, chrysene, naphthalene) as well as historically underrepresented nitrogenated (NPAHs), oxygenated (OPAHs), and alkylated (APAHs) PAH homologs (primarily in the C_10_–C_25_ range). Method detection limits (MDLs) have also improved considerably such that levels of UPAHs and substituted PAHs can now be determined in the low parts per billion to high parts per trillion (Gohlke et al. 2011; Overton et al. 2004; Rotkin-Ellman et al. 2011; Xia et al. 2012). These values are often 1–3 orders of magnitude lower than levels of health concern for the small subset of PAHs for which we have adequate toxicological and health risk information.

UPAHs such as benzo[*a*]pyrene are primarily produced through incomplete combustion or the pyrolysis of organic material, including fossil fuels. NPAHs and OPAHs are produced through combustion of fossil fuels, atmospheric processes, and microbial and enzymatic activity (Albinet et al. 2007; Durant et al. 1996; Lundstedt et al. 2007). APAHs are generally found in relatively high concentrations as native constituents in crude oil or petroleum and have been used as petrogenic biomarkers to identify unrefined, uncombusted petroleum in the environment (Overton et al. 2004; Saha et al. 2009). Overton et al. (2004) used GC/MS-SIM to apportion PAH sources in coastal, marine sediments as a function of historical and contemporary oil and gas activity in the Gulf of Mexico, categorically discriminating the pyrogenic PAHs (e.g., naphthalene) from the petrogenic PAHs (e.g., methylnaphthalenes).

The toxicology of several of the UPAHs has been extensively studied. Within the class of PAH compounds, there are both noncarcinogens and carcinogens. In general, the small PAHs (2- to 3-ring members) act as noncarcinogens that mainly affect the respiratory, neurological, or immune system. At high concentrations, some of the smaller PAHs may act as comparatively weak carcinogens. The larger PAHs (4- to 7-ring members) may also act as noncarcinogens (e.g., immunotoxic), but they act primarily as fairly potent carcinogens through a mutagenic mode of action (Agency for Toxic Substances and Disease Registry 1995; International Agency for Research on Cancer 1973, 1983; National Toxicology Program 2011). Based on current knowledge, the various PAHs found in environmental media, including seafood, and associated with either noncancer health effects or cancer are present in varying concentrations. The levels of concern (LOCs) for PAHs that may cause noncancer health effects are considerably higher than those for PAHs that are linked to cancer (Gohlke et al. 2011; Rotkin-Ellman et al. 2011). For example, in shrimp or other orally consumed media, the LOCs (i.e., concentration at which an adverse health effect may be expected for a fraction of the exposed population) for noncancer health effects expected from PAHs (naphthalene, anthracene, 2-methylnaphthalene, and acenaphthene) range from low to high parts-per-million levels (Gohlke et al. 2011; Rotkin-Ellman et al. 2011). In contrast, for cancer effects expected from the PAH benzo[*a*]pyrene, the LOC is in the low parts-per-billion range (Gohlke et al. 2011; Rotkin-Ellman et al. 2011).

The levels of PAHs detected in seafood and many other environmental media are often low (not detected to low parts per billion) and are either below or near the LOCs for cancer health risks (Gohlke et al. 2011; Rotkin-Ellman et al. 2011; Xia et al. 2012). Some food items (e.g., smoked foods) have comparatively high levels of PAHs, mostly higher-molecular-weight compounds, on their surface; these PAHs may represent both noncancer risks and cancer risks (Silva et al. 2011; Stolyhwo and Sikorski 2005). PAHs in seafood tested during and after the *Deepwater Horizon* event, up to the present day, were at or below the LOCs for cancer health risks and far below those associated with noncancer health risks. This is the case for PAHs found in most environmental media, including foodstuffs. Thus, when considering the vast majority of human population exposures to PAHs in environmental media, the primary health concern based on the toxicological evidence to date is cancer. Some researchers have acknowledged cancer as the primary health concern following the *Deepwater Horizon* incident, along with showing concern about possible contamination of seafood (Dickey 2012; Gohlke et al. 2011; Rotkin-Ellman and Solomon 2012; Rotkin-Ellman et al. 2011; Xia et al. 2012). Therefore, we will address PAH exposures from the perspective of carcinogenesis.

UPAHs generally require enzymatic bioactivation to be converted to highly reactive compounds that covalently modify DNA, forming premutagenic DNA lesions or adducts (Klaunig and Kamedulis 2008; Shimada and Fujii-Kuriyama 2004). A few of the NPAHs and OPAHs have also been evaluated for mutagenic potency, and many of these exhibit genotoxicity with or without enzymatic activation (Durant et al. 1996). The UPAHs show considerable heterogeneity in mutagenic and hence carcinogenic potency [Collins et al. 1998; Nisbet and LaGoy 1992; U.S. Environmental Protection Agency (EPA) 2000, 2010], which is thought to derive from the structural properties of the various UPAHs. Genotoxicity appears to be a function of bay or fjord configurations in which the presence and size of the configuration influences the detoxification efficiency of the bioactivated metabolites. For example, naphthalene is relatively nongenotoxic and has neither a bay or fjord configuration or moiety. On the other hand, benzo[*a*]pyrene and dibenzo[*a,i*]pyrene are genotoxic and carcinogenic UPAHs with different individual potencies that harbor a bay and fjord region, respectively.

The levels of UPAHs in environmental media and food are monitored by federal health agencies including the U.S. EPA, FDA, and National Oceanic and Atmospheric Administration (NOAA). However, compared with the APAHs, the UPAHs comprise a relatively small fraction of the total number and mass of PAHs found in crude oil and crude oil–contaminated seafood (Saha et al. 2009). Xia et al. (2012) examined seafood from areas affected by the *Deepwater Horizon* incident for PAHs, including APAHs; they found that low-molecular-weight UPAHs and several APAHs were present in most seafood types examined. UPAHs used in health risk assessments conducted by the FDA and others were generally low or below detection limits (Dickey 2012; Rotkin-Ellman et al. 2011; Xia et al. 2012). Xia et al. (2012) also noted higher levels of total PAHs during July–October 2010, which fell to much lower levels by early 2011. It is plausible that APAHs were in some measure responsible for the higher levels of total PAHs during the spill. But it is unfortunate that comparatively few of the APAHs have been evaluated for toxicity or mutagenicity. Results for the APAHs that have been evaluated suggest reasons for concern. For example, 5-methylchrysene is significantly more toxic and carcinogenic than is the unsubstituted parent chrysene (Hecht et al. 1978). The lack of toxicological data and related risk information on the APAHs represents an especially critical gap in the scientific data because, by mass, the APAHs constitute the vast majority of PAHs in crude oil and petroleum with the potential to contaminate seafood following a marine spill event (Baird et al. 2007).

An important opportunity exists for narrowing the knowledge gap between what researchers currently know about specific types and levels of PAHs in seafood and how that information can be used to estimate possible increases in health risks following consumption. The U.S. EPA currently includes 16 UPAHs in the analysis of environmental media (i.e., soil/sediment, water, or air) for protecting public health (Nisbet and LaGoy 1992; Schoeny and Poirier 1993). Seven of these UPAHs are considered key carcinogens for the purposes of policy-based cancer risk assessments (Schoeny and Poirier 1993; U.S. EPA 2000). With the exception of benzo[*a*]pyrene, oral slope factors for estimating cancer risks are unavailable at the federal level for most of these recognized carcinogens. Therefore, researchers and the U.S. EPA have developed and applied relative potency factors designed to scale carcinogenic potencies to benzo[*a*]pyrene assuming simple additive toxicity (Collins et al. 1998; Nisbet and LaGoy 1992; U.S. EPA 2000, 2010). The FDA and NOAA risk assessment protocols include the 16 U.S. EPA PAHs and 9 additional PAHs, including a select few APAHs (FDA 2010). These protocols are designed to provide quantitative measurements for assessment of ingestion health risks and inform any risk management strategies or consumption advisories that may be warranted. In addition, the FDA and NOAA protocols can provide limited information on the source of the PAHs (i.e., pyrogenic or petrogenic).

Research scientists currently funded to conduct seafood safety assessments in response to the *Deepwater Horizon* accident are now including an additional 25–50 PAHs, most of which are APAHs, to better define the pyrogenic versus petrogenic origin of these compounds in seafood and marine species (e.g., Xia t al. 2012). These consortia, with whom many of us are working, are in the process of collecting and analyzing seafood, so that scientific data has not yet been published. Thus, this emerging gap in risk assessment is driven by *a*) the contemporary technological capacity to measure an array of PAHs at extremely low concentrations, and *b*) the general absence of toxicological information for many PAHs and virtually all of the APAHs regarding toxicity, mutagenicity, or carcinogenicity. Closing this gap should be a pressing concern for scientists, risk assessors, and public health officials. Our ability to quantify 50 to hundreds of PAHs—while having meaningful information on human health risk for only a few of them—is a major problem for conducting risk assessments and accurately communicating risks regarding seafood safety and other situations in which substituted PAHs represent either consumption or inhalation risks.

Our capacity to detect smaller and smaller quantities of PAHs in seafood and other matrices could lead to misperceptions by the public about the health risks that they may face after oils spills and natural disasters. Risk assessments often treat nondetects or levels below the MDL as zero when evaluating health risks. The lay public may interpret such nondetect or sub-MDL findings in seafood samples as indicating that they are essentially free of PAHs. Today, however, many more PAH analytes can be assigned quantitative values in complex environmental media, such as seafood, that may have previously been considered PAH free. This means that analytes with quantitative values above the currently available MDLs (low parts per billion/high parts per trillion) can be used in mixtures risk assessment models, even if individual PAHs are present in concentrations far below currently accepted levels of health concern. This new analytical capacity may trigger health concerns among members of the general public, for whom the mere detection of PAHs in seafood may be interpreted as evidence of a problem. Adding complexity to this emerging problem is the relative absence of a comprehensive evidence base by which toxicity, mutagenicity, or carcinogenicity can be assigned to the growing list of analytes that researchers are now able to detect. This is especially true for the APAHs, which, in oils and uncombusted fuels, represent the majority of PAH contaminants resulting from environmental spills and accidents (Baird et al. 2007; Saha et al. 2009; Xia et al. 2012). How then do we deal with more sensitive and comprehensive results and the increased concerns that they may elicit in the general public? Should monitoring efforts simply avoid evaluating the levels of this growing list of PAHs, including APAHs, until they can effectively be used in policy-based public health protection and management strategies?

## Conclusions

The questions and issues raised here represent deficiencies that the entire cadre of stakeholders, including the affected public, research scientists, public health officials, medical professionals, funding agencies, and industries, can help address. Investment is needed in both research and education to fill the gaps in critical knowledge and potential public perception. Investments in environmental education and literacy will give stakeholders a better understanding of why an expanded list of PAH analytes, including APAHs, is warranted. In addition, stakeholders will be better able to understand what the various quantitative levels of PAHs mean from a point of evidence-based public health protection and gain a greater appreciation for the value of the health risk assessment process, including its limitations. Achieving these end results will better engage affected communities, improve the application and use of resources in the areas most impacted, and foster more effective risk communication and information dissemination strategies. These goals can be achieved with investments in improving analytical chemical methods, toxicology, and risk assessment research to develop the evidence base required to objectively evaluate relevant PAHs, including the APAHs, in the health risk assessment process.

Currently, there are both government and academic laboratories with the requisite expertise necessary to perform the critical experimentation required to assign toxicity and related risk values to the relevant compounds (e.g., studying bay and fjord region APAHs to evaluate mutagenicity and mechanistic differences relative to their respective UPAHs). High-throughput screening methods (e.g., *in vitro* reporters or cell systems, the Tox21 initiative) could be used to identify relevant compounds for more detailed experimentation and analysis (e.g., animal toxicity, carcinogenicity using defined mixtures). Scaling or potency factors, as well as risk values for defined mixtures themselves, can be developed from this work (U.S. EPA 2000, 2010). Time and resource requirements will no doubt be substantial, but this should be a major topic of discussion with relevance to not only PAHs but other large classes of chemical compounds that largely remain untested for toxicity. We cannot test each and every PAH independently and in the exponential number of combinations and concentrations possible. Environmental analyses determining the detectable levels and types of PAHs present in a medium of concern should be used to prioritize testing. APAHs of highest abundance in the media of concern should receive higher priority ranking. Furthermore, the APAHs whose parent compound or UPAH is carcinogenic should receive high priority with respect to toxicology testing designed to address cancer risks. Specifically testing APAHs in which the alkyl side groups modify the characteristics of a bay or fjord region should be considered a priority. We have only briefly mentioned some general approaches that should be part of the conversation. The National Toxicology Program uses a number of approaches designed to determine adverse effects of chemicals, including genotoxicity and carcinogenicity. These encompass new high-throughput initiatives such as Tox21 and the well-standardized 2-year rodent bioassay (Tice et al. 2013). These approaches should be used where appropriate. *In vitro* methods, such as high-throughput aryl hydrocarbon receptor binding assays or the CALUX® assay, could be used as screening tools. *In silico* tools will no doubt be helpful, but these digital models require bench-derived data to be most informative. No single method or assay is likely to be sufficient in terms of both accuracy and precision. Finally, well-informed and designed whole-organism experiments (e.g., rodent bioassays) will still be necessary because they better capture the relevant exposure(s) and dose responses in bioactivation, detoxification, mutagenesis, genome maintenance/dysregulation, and ultimately cancer. One system that may be useful is a high-throughput zebrafish system to test for developmental toxicity for a variety of OPAHs (Knecht et al. 2013). Systems for evaluating tumor formation (e.g., the *Xiphophorus* fish model; Walter and Kazianis 2001) would be useful for rapid testing. Care must be taken to ensure that any model system developed for evaluating the carcinogenic potency of APAHs accurately models humans in terms of exposure and dose response as well as cancer. We contend that *in vitro* and cell-based systems alone do not provide the necessary entire context that whole-animal studies provide.

Through a concerted effort, toxicologists, biochemists, analytical chemists, health risk researchers, and community-based researchers working with policy makers can provide the evidence base from which to effectively translate key findings into public health protection policy. Such policy implementation will itself require considerable time and debate among all stakeholders. However, having an adequate evidence base in place to inform policy making will improve and modernize efforts directed toward mitigating public health and economic impacts caused by future petroleum spills.
